# Trypanocidal Mechanism of Action and *in silico* Studies of *p*-Coumaric Acid Derivatives

**DOI:** 10.3390/ijms20235916

**Published:** 2019-11-25

**Authors:** Susiany P. Lopes, Yunierkis P. Castillo, Marilia L. Monteiro, Ramon R. P. P. B. de Menezes, Reinaldo N. Almeida, Alice M. C. Martins, Damião P. de Sousa

**Affiliations:** 1 PostGraduation Program in Technological Development and Innovation in Medicines, Federal University of Paraíba, João Pessoa CEP 58051-970, Paraíba, Brazil; susiany_lopes@hotmail.com; 2 Escuela de Ciencias Físicas y Matemáticas, Universidad de Las Américas, Quito 170125, Ecuador; yunierkis@gmail.com; 3 Department of Clinical and Toxicological Analysis, Federal University of Ceará, Fortaleza CEP 60020-181, Ceará, Brazil; marilialopesmonteiro@yahoo.com.br (M.L.M.); ramonppessoa@ufc.br (R.R.P.P.B.d.M.); martinsalice@gmail.com (A.M.C.M.); 4 Postgraduate Program in Natural and Synthetic Bioactive Products, Federal University of Paraíba, João Pessoa CEP 58051-970, Paraíba, Brazil; reinaldoan@uol.com.br; 5 Department of Pharmaceutical Sciences, Federal University of Paraíba, João Pessoa CEP 58051-970, Paraíba, Brazil

**Keywords:** natural products, esters, cinnamic acid, phenylpropanoid, *Trypanosoma*, neglected diseases, antiparasitic activity

## Abstract

*Trypanosoma* species are responsible for chronic and systemic infections in millions of people around the world, compromising life quality, and family and government budgets. This group of diseases is classified as neglected and causes thousands of deaths each year. In the present study, the trypanocidal effect of a set of 12 ester derivatives of the *p*-coumaric acid was tested. Of the test derivatives, pentyl *p*-coumarate (**7**) (5.16 ± 1.28 μM; 61.63 ± 28.59 μM) presented the best respective trypanocidal activities against both epimastigote and trypomastigote forms. Flow cytometry analysis revealed an increase in the percentage of 7-AAD labeled cells, an increase in reactive oxygen species, and a loss of mitochondrial membrane potential; indicating cell death by necrosis. This mechanism was confirmed by scanning electron microscopy, noting the loss of cellular integrity. Molecular docking data indicated that of the chemical compounds tested, compound **7** potentially acts through two mechanisms of action, whether by links with aldo-keto reductases (AKR) or by comprising cruzain (CZ) which is one of the key *Trypanosoma cruzi* development enzymes. The results indicate that for both enzymes, van der Waals interactions between ligand and receptors favor binding and hydrophobic interactions with the phenolic and aliphatic parts of the ligand. The study demonstrates that *p*-coumarate derivatives are promising molecules for developing new prototypes with antiprotozoal activity.

## 1. Introduction

Neglected diseases are a group of diseases which compromise quality of life and bring death to a considerable part of the world’s population, especially in underdeveloped and developing countries [1,2]. It is estimated that these diseases affect more than one billion people around the world [3,4,5]. Strategies to control or eliminate these diseases through interventions, such as vector control and neutralization of the infectious agent are of great importance [6,7]. American trypanosomiasis or Chagas’ disease is caused by the parasite *Trypanosoma cruzi* (*T. cruzi*) [8,9,10,11]. Currently 6 to 8 million people are infected and more than 100 million are at risk [12]. Chagas’ disease (CD) presents two clinical stages, an acute phase where symptoms are more asymptomatic, and a chronic phase which leads to heart failure or even death [13]. Currently, two drugs are used against the disease; benznidazole and nifurtimox [14]. Both present side effects ranging from allergic reactions, to fever, insomnia, and also gastrointestinal symptoms such as weight loss and nausea [15,16]. Despite efforts to develop an effective and safe therapeutic approach, there are still no effective vaccines [17,18,19]. Because of this, the search for new effective and safe compounds to treat Chagas disease has been the aim of various studies [20].

Natural products used in treating this type of infection are promising [21,22]. For example, hydroxycinnamic acid presents various pharmacological activities: anti-inflammatory, antimicrobial, antioxidant, antitumor, and antiviral [23,24]. Hydroxycinnamic acids are found in much of the human diet such as caffeic acid (CaA), *p*-couramic acid (*p*CoA), and ferulic acid (FeA). They are found in wine, vegetables, fruits, seeds, and sugarcane-sugar [25]. *p*-Coumaric acid is a phenolic secondary metabolite abundant in nature [26,27,28,29,30]. It is of great importance for presenting antioxidant, anti-inflammatory, anticancer, chemo-protectant, anti-diabetic, and antimicrobial properties [31]. Derivatives of *p*-coumaric acid, amide N-*trans*-*p*-coumaroylthyramine and its analogues, were isolated from a bioactive methanolic extract of *Polygonum hyrcanicum* in a biomonitoring study and presented trypanocidal activity against *Trypanosoma brucei* [32]; *p*-coumaric acid, also isolated from the extract, presented lower trypanocidal potency.

Thus, given reports of trypanocidal activity for hydroxyl benzoic derivatives, and due to the lack of studies in the literature concerning the trypanocidal activity of *p*-coumaric acid derivatives, as well as the therapeutic potential of this chemical class against *Trypanosoma* species, the proposal of this study was to prepare and evaluate the trypanocidal activity of a collection of *p*-coumaric acid derivatives.

## 2. Results and Discussion

### 2.1. Chemistry of Compounds **1–12**

**Scheme 1:**
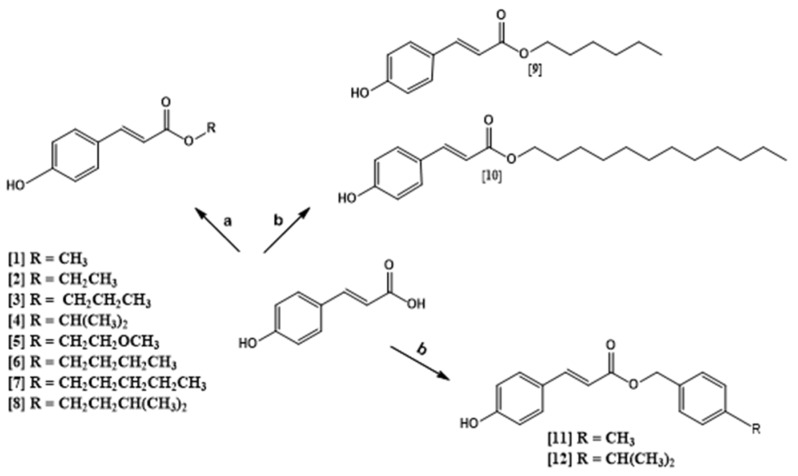
Preparation of the *p*-coumaric acid derivatives: (**a**) ROH, H_2_SO_4_, reflux; (**b**) ROH, THF, TPP, DIAD, 0 °C to r.t.

In this work, twelve compounds were prepared, maintaining the (*E*)-3-(4-hydroxyphenyl) acrylic acid structure and modifying only the radical *R* for: Methyl (**1**), Ethyl (**2**), Propyl (**3**), Isopropyl (**4**), Methoxyethyl (**5**), Butyl (**6**), Pentyl (**7**), Isopentyl (**8**), Hexyl (**9**), Dodecyl (**10**), 4-Methylbenzyl (**11**), and 4-Isopropylbenzyl (**12**) esters. In the derivatives reaction, (*E*)-3-(4-hydroxyphenyl) acrylic acid and ROH were used. Most of the products obtained a good yield and the reactions were carried out in a single step. Compounds **1–8** were prepared by Fischer esterification, the products were obtained within 5–27 h, presenting satisfactory yields (34.50–90.63%). For compounds **9–12** the Mitsunobu reaction was used, and reactions times were around 72 h with yields of 29–50% (Scheme 1).

The infrared spectra of the *p*-coumaric acid analogs presented absorption bands above 3000 cm^−1^ relative to stretch C-H sp^2^, C = C stretch ring absorption bands occurring in pairs in the 1600 and 1475 cm^−1^ regions, C = O stretch bands occurred in the range 1750–1730 cm^−1^, the C-O stretch assigned to the ester bond appeared with two bands in the range 1300 to 1000 cm^−1^. Alkyl substituents show C-H (sp^3^) stretch bands at about 3000 cm^−1^, strain absorbing methylene groups at 1465 cm^−1^, and methyl groups at 1375 cm^−1^. Regarding hydroxyl binding to the aromatic ring, it presented a stretch peak in the 3400–3200 cm^−1^ region.

The ^1^H and ^13^CNMR spectra of the synthesized products demonstrate the presence of six common hydrogens (H-2 and H-6, H-3 and H-5, H-7, H-8) being four aromatic ring hydrogens, and two side chain hydrogens; as well as nine carbons in common (C-1, C-2, C-3, C-4, C-5, C-6, C-7, C-8, C-9), being six from the aromatic ring and three from the side chain with the presence of carbonyl that confirms the structures. The hydrogen signals obtained in (CDCl_3_, 400 MHz, ppm), with greater displacement, related to compound **12** were: for olefinic hydrogen we have two doublets at δ 7.69 (*d*, *J* = 15.96 Hz, 1H) and 6.35 (*d*, *J* = 15.96, 1H), respectively assigned to the H-7 and H-8 hydrogens, where the trans configuration is confirmed by the coupling constant (J = 15.96 Hz). For aromatic hydrogens we have two doublets at δ 7.42 (*d*, *J* = 8.4, 2H) and 6.87 (*d*, *J* = 8.55, 2H) assigned to protons H-2 and H-6; H-3 and H-5, respectively. Similar coupling constants highlight the coupling that occurs between neighboring hydrogens and the signals of the methylene and methyl saturated side chain hydrogens, as well as the methoxy signals of aromatic hydrogens.

^13^CNMR data present chemical shift values that also confirm the identity of the compounds. For ^13^CNMR spectra, the signals obtained at (CDCl_3_, 100 MHz, ppm), with greater displacement, for compound **8** were: 168.6 characteristic of ester C = O, and 145.1 attributed to carbon C-7, 158.6 of C-4, 126.8 of C-1, 115.2 of C-8, and signals in the region of 130.2 to 116.1 assigned to the carbons of the *p*-coumaric ring, respectively C-2 and C-6, C-3 and C-5. In addition to these signals, chemical shifts of saturated and aromatic side chain carbons were also evidenced, confirming the structure of each compound.

### 2.2. Trypanocidal Activity of Compounds **1–12**

The trypanocidal activity of the *p*-coumaric derivatives (**1–12**) were evaluated against epimastigotes and trypomastigote forms of *T. cruzi* (strain Y) at 24 h of incubation and in host LLC-MK2 cells at 24 h of exposure to different concentrations: 100; 50; 25; 12.5; 6.25; 3.12; and 1.56 μg/mL. In the MTT assay it was possible to calculate the IC_50_ of the tested substances through cell viability, which was calculated in relation to the negative control, whose absorbance was considered 100%. The results were evaluated as IC_50_ and measured in μM as shown in the Table 1.

### 2.3. Cell Death Profile

Epimastigote forms were used to assess the cell death profile using labeling with AxPE and 7-AAD. For data interpretation, the cells were divided into four cell populations: viable cells, with low levels of labeling for both fluorochromes (un-labeled); necrotic cells, labeled only with 7-AAD (7AAD +); apoptotic cells, labeled with annexin V-PE only (/ AxPE +); and doubly labeled late apoptotic cells (7AAD +/AxPE +) [33].

**Figure 1:**
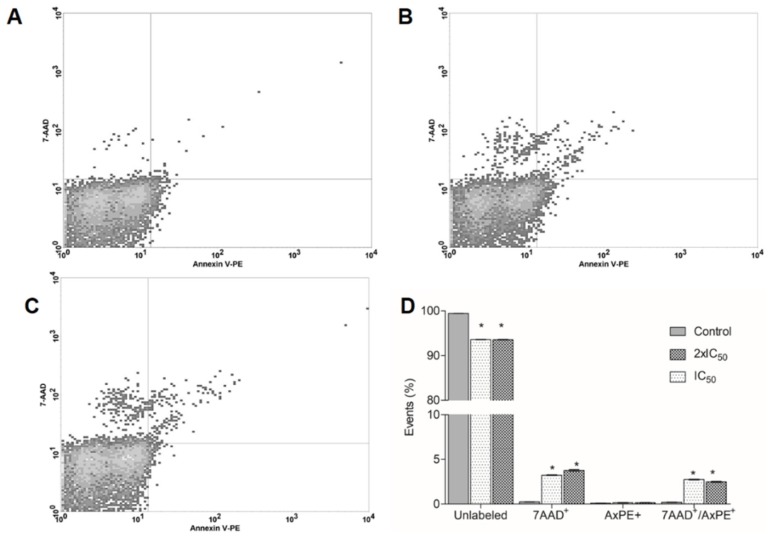
7 AAD and annexin V-PE labeling tests. Density figure presentations (**A**–**C**) of control (**A**), (**B**) with derivative 7 on BIS 5.164 μM, and (**C**) at 10.329 μM. In the lower left quadrant, viable cells are represented; while in the upper left quadrant are 7AAD + population (necrosis); in the lower right quadrant ha Ax + population (early apoptosis); and right upper quadrant corresponds to population 7AD/Ax + (late apoptosis); (**D**) percentage numbers of cells (marked and unmarked) in the evaluation. Results are shown as mean ± SEM of tests performed in triplicate. * *p* < 0.05 vs. control group.

Cells were incubated with compound **7** for 24 h at concentrations of 5.164 and 10.329 μM, equivalent to IC_50_ and 2 × IC_50_. In this assay, a reduction in the percentage of viable cells and an increase in the percentage of 7-AAD-labeled cells was observed, indicating cell death by necrosis (Figure 1A–C). Necrosis results in damage to the plasma membrane and cell organelles [34]. The increase is also observed in the displacement of cell populations, as shown in Figure 1D, which contains the scatter plot density graphs of the cells.

### 2.4. Analysis of Reactive Oxygen Species

**Figure 2:**
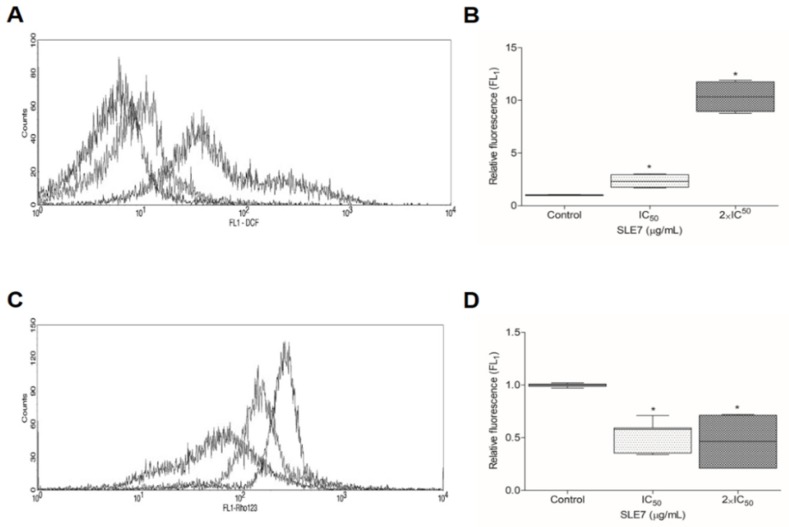
Flow cytometry (FC) evaluations for cytocytic measurement of ROS and mitochondrial transmembrane potential (ΔΨm). (**A**) FC histogram overlap of cultures treated with derivative **7** after 24 h incubation for DCF labeling; (**B**) bending alteration in geometric means in DCF; (**C**) shows FC histogram overlap of derivative **7** treated cultures for Rho123 labeling after 24 h incubation; (**D**) Fold-change in geometric means on Rho123. The data are presented as mean ± SEM of three independent experiments performed in triplicate. * *p* < 0.05 vs. control group.

Figure 2A,B presents the effect of compound **7** on the production of reactive species in *T. cruzi* epimastigote forms. A relative fluorescence increase was observed in both treated groups as compared to the control. The results indicate that with increased reactive oxygen species, compound **7** induces oxidative stress on the parasite. Thus, flow cytometry analysis was performed to verify the ability of compound **7** to change *T. cruzi* parameters [35].

### 2.5. Mitochondrial Transmembrane Potential

When evaluated with rhodamine 123, cells treated with compound **7** presented a reduction in rhodamine accumulation, indicating mitochondrial injury. This helps explain the cell death of the parasite [36,37]. Figure 2C,D illustrate the results.

### 2.6. Scanning Electron Microscopy

**Figure 3:**
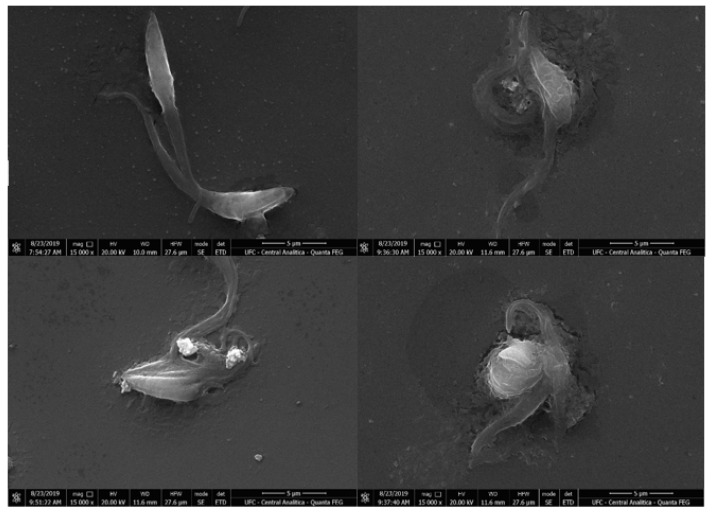
Scanning electron microscopy images of *Trypanosoma cruzi* epimastigotes. Untreated epimastigotes (control; (**A**)), epimastigotes treated with IC_50_ (**B**,**C**) and 2 × IC_50_ for compound **7** (**D**). Treated parasites showed ultrastructural changes, such as changes in typical shape, apparent leakage of cytoplasmic content, and cell membrane degradation. Scale bar =5 μm.

Morphological changes in epimastigote forms induced by compound **7** after 24 h of treatment were analyzed by scanning electron microscopy. Ultrastructural changes were observed in the treated groups, such as changes in typical shape, apparent leakage of cytoplasmic content, and cell membrane degradation as shown in Figure 3 [38].

The analysis of the *p*-coumaric acid derivatives was based on 50% inhibitory concentration (IC_50_) results for the trypanocidal activities tested. Twelve compounds were tested for trypanocidal activity against *T. cruzi* epimastigotes, trypomastigotes, and LCC-MK2 cells. Of the collection presented in Table 1, four presented good activity against epimastigote forms (compounds 3, 5, 7, and 8).

We observed that methyl *p*-coumarate, compound (**1**) presented the lowest antiparasitic potency, with a high IC_50_ (601.06 ± 249.17 μM). On the other hand, substitution with an ethyl group in compound **2** resulted in a 50% decrease in inhibitory concentration (IC_50_) (216.06 ± 109.25 μM) when compared to compound **1 [39]**. These same characteristics were observed against the *Trypanosoma brucei* species in a study by Taladriz et al. (2012) [40]. Corroborating these results, Lima et al. (2015) showed that ethyl caffeate (18.27 μM) presents good activity against *T. cruzi* amastigotes [41].

When we analyzed compounds **3**, **4**, and **5**, all (with three-carbon side chains) presented a difference in their IC_50_ values (16.76 ± 1.93 μM, 334.90 ± 54.30 μM and 32.03 ± 5.39 μM, respectively). We observe that modification of the n-propyl radical (**3**) to isopropyl (**4**) contributes to a reduction in activity, possibly due to the volume of the isopropyl group that compound **4** presents in its branch. Propyl *p*-coumarate (**3**) was twice as potent as 2-methoxyethyl *p*-coumarate (**5**) [42,43]. Compound **3** also presented greater selectivity than compound **5** for the epimastigote form (SI = 26.55, SI ≥ 14.05, respectively), and the trypomastigote form (SI = 3.8, SI ≥ 1.25, respectively). This is probably due to the presence of oxygen in the aliphatic chain, decreasing trypanocidal activity [44].

Compound **6** presented an IC_50_ of 154.99 ± 33.14 μM; an increase in trypanocidal potency as compared to compound **1**, this is possibly related to the lipophilicity of the derivative, increasing its penetration across the parasite cell membrane, and consequently influencing the parasite cell membrane and increasing trypanocidal activity [45,46].

Of the derivatives evaluated, pentyl *p*-coumarate (**7**) and isopentyl *p*-coumarate (**8**) were the compounds with the lowest IC_50_ values, both in epimastigote forms (5.16 ± 1.28, and 13.23 ± 2.56 μM, respectively), and in trypomastigote forms (61.63 ± 28.59, and 111.31 ± 38.70 μM, respectively). Compound **7** when compared to compound **8** had a higher selectivity index in the epimastigote form (SI = 23.86, SI = 9.1, respectively), than in the trypomastigote form (SI = 2, SI = 1.08, respectively). This explains the high antiparasitic activity against the epimastigote form. Compound **7** was considered the most potent of the derivatives analyzed. This compound presents hydrogen bonds with the receptor, as well as hydrophobic and van der Waals bonds with the phenolic and aliphatic parts and is able to block the activity of the *T. cruzi* enzymes CZ and AKR, as shown in the molecular docking study. Studies conducted by Steverding et al. (2016) found that isopentyl caffeate, which was used for its activity against *Trypanosoma brucei*, inhibited parasite growth. Thus, five-carbon alkyl chains on hydroxycinnamic derivatives should contribute to trypanocidal activity [47].

Compounds **9** (68.86 ± 18.12 μM) and **10** (50.55 ± 18.94 μM) presented higher IC_50_ values as compared to compound **7**. This is possibly due to carbon chain increases to six and twelve carbons, thus observing a decrease in trypanocidal derivatives activity [48]. With respect to compounds **11** (75.47 ± 13.41μM) and **12** (68.86 ± 18.12 μM) which did not show significant trypanocidal activity. Replacing an aliphatic chain with an aryl radical resulted in decreased antiparasitic potency [49].

Considering the results presented by compound **7**, and due to its high selectivity, we analyzed its antiparasitic mechanism of action against the epimastigote form. In flow cytometric analyses dealing with compound **7**, a reduction in the percentage of viable cells and an increase in the percentage of 7-AAD-labeled cells was observed, indicating cell death by necrosis [50]. An increase in relative fluorescence was observed at both tested concentrations of compound **7** (5.164 μM, and 10.329 μM), as well as an increase in production of reactive oxygen species, inducing oxidative stress against the parasite. Thus, cells treated with compound **7** presented a reduction in the accumulation of rhodamine 123, causing loss of mitochondrial transmembrane potential [51,52]. Upon analysis of the images performed by scanning electron microscopy, alterations and loss of cellular integrity were observed in the groups treated with compound **7**; this was confirmed by the flow cytometry data [53]. The data corroborate previous studies, where rupture of *T. cruzi* trypomastigote plasma membranes when using the *p*-coumaric derivative [38] was observed.

### 2.7. Computational Methods

From the consensus computational target fishing approach three families of potential targets in homo sapiens were identified: Carbonic anhydrases, aldo-keto, and aldose reductases and the hydroxycarboxylic acid receptor 2. Among them, only carbonic anhydrases (CA) and aldo-keto reductases (AKR) were found to have orthologous proteins in *T. cruzi* and they were considered for docking studies with compound **7**. Among all three target fishing approaches, only one protein from *T. cruzi* (glyceraldehyde-3-phosphate dehydrogenase, GAPDH) was identified as potential target for the query compound. Although this prediction received a low support from SEA, GAPDH was also selected for modeling studies. In addition, set of known anti-trypanosomal targets comprising cruzain (CZ), mitochondrial iron-dependent superoxide dismutase (SOD-A), cytoplasmatic iron-dependent superoxide dismutase (SOD-B) and trypanothione reductase (TR).

Consequently, compound **7** was docked to below listed potential targets following the procedure described in the Computational Methods section. The results of the molecular docking calculations are summarized in Table 2. For GAPDH it has been observed that ligand can bind in presence of the NAD+ cofactor or it can displace the cofactor from its binding site and occupy the newly vacant region [54,55]. For this reason, binding hypotheses for compound 7 were generated in the presence of NAD+ and with absent cofactor.

As can be seen from Table 2, only one probable binding mode of compound 7 was predicted for the CA, SOD-B and GAPDH+NAD targets. In contrast, three potential binding modes were predicted to AKR and GAPDH, while two probable binding modes were predicted for CZ, SOD-A, and TR. The predicted binding pose of compound **7** to CA resembles that of ligands to the orthologous enzyme in homo sapiens [56] and it is provided as Supporting Information in Figure S1. No hydrogen bond between the ligand and the receptor is predicted to stabilize the complex. However, the ligand makes an extensive network of contact with the receptor. The pentyl moiety accommodates in a hydrophobic cavity flanked by L139, V127, V141, L203, V212, and F214. In addition, the carbonyl group points to the region containing the histidine residues that coordinate the zinc atom and the phenyl moiety make contacts with non-aromatic hydrophobic residues while it is exposed to the solvent.

The three probable binding poses of compound 7 to AKR also share the same binding region as its human counterpart inhibitors [57,58]. These predicted binding modes are shown in Supporting Information Figures S2–S4. As for the homo sapiens AKR inhibitors, the three predicted conformers of compound **7** occupies the enzyme active site blocking the access to the NAP+ cofactor.

Conformers 1 and 3 are predicted to interact through hydrogen bonds with W113 (conformer 1) and H112 (conformer 2). These interactions take place through the ligand phenol and carboxy groups, respectively. The predicted conformers also make extensive contacts with the receptor that involve NAP+, W25, R26, I53, Y54, S55, W81, H112, W113, W189, S194, I270, G271, and F277.

In the case of CZ, both predicted binding poses of compound **7** are in agreement to that observed for the known CZ inhibitors [59] as represented in Supplementary Information Figures S5 and S6. These binding modes are predicted to form no hydrogen bonds with the receptor, however, both conformers make contact with the catalytic C25 residue. Furthermore, the predicted conformers interact with G23, S64, G65, G66, L67, M68, N69, N70, A138, D161, H162, and E208.

In both the highly homologous SOD-A and SOD-B enzymes, compound **7** binds close to their catalytic site located in the dimer interface as shown in Supporting Information Figures S7–S9. None of the two binding poses predicted for SOD-A interact through hydrogen bonds with the receptor. They also differ in the orientation of their aliphatic chains while the phenol moiety positions favorably to contact F327 of the other subunit through π-stacking. Compound **7** bound to SOD-A also interacts with residues from both chain in the dimer that include K32, H33, A36, K40, and Q73 from the first monomer as well as V324, N325, E370, N379, R381, and P382 from the second subunit. On the other hand, the compound under investigation is predicted to form two hydrogen bonds with H31 of the first monomer and with E355 of subunit B through the hydroxyl group of the phenol moiety. In addition, compound **7** makes an extensive network of contacts with SOD-B through Y35, K38, P64, L67, N68, and H163 of the first monomer and G310, F312, G313, and R366 of the second subunit.

The two probable conformers of compound **7** bound to TR share the same binding pocket as the previously identified TR inhibitors [60]. Conformer 1 is stabilized by a hydrogen bond between the hydroxyl group of the phenol moiety to S10 while the later stacks below W17 in conformer 2 as shown in Supporting Information Figures S10 and S11. In addition to S10 and W17, both conformers interact with G9, L13, E14, G45, C48, V49, Y106, M109, T330, and I334. In contrast to the rest of the identified potential targets for compound 7, its predicted binding poses to GAPDH shows that the most probable conformers of the compound to this enzyme is located away from the known binding site of its substrate and enzyme inhibitors. The later observation holds for the receptors containing NAD and depleted of the cofactor as can be seen in the Supporting Information Figures S12–S15. Thus, compound **7** is unable to block the binding of substrates to GAPDH and it will be discarded for further investigations.

Hitherto, all the predicted binding modes of compound **7** to the assayed targets, except for GADPH, are plausible. To get more insights into these predicted complexes, were performed molecular dynamics simulations and MM-PBSA calculations for them as described in the Computational Methods section. The predicted free energy of binding of compound **7** to its potential targets is summarized in Table 3 and the only favorable values of binding energy are obtained for the targets AKR and CZ. Based on these results, were performed further analyses to the predicted binding modes of compound **7** to AKR (conformer 3) and CZ (conformer 1).

**Figure 4:**
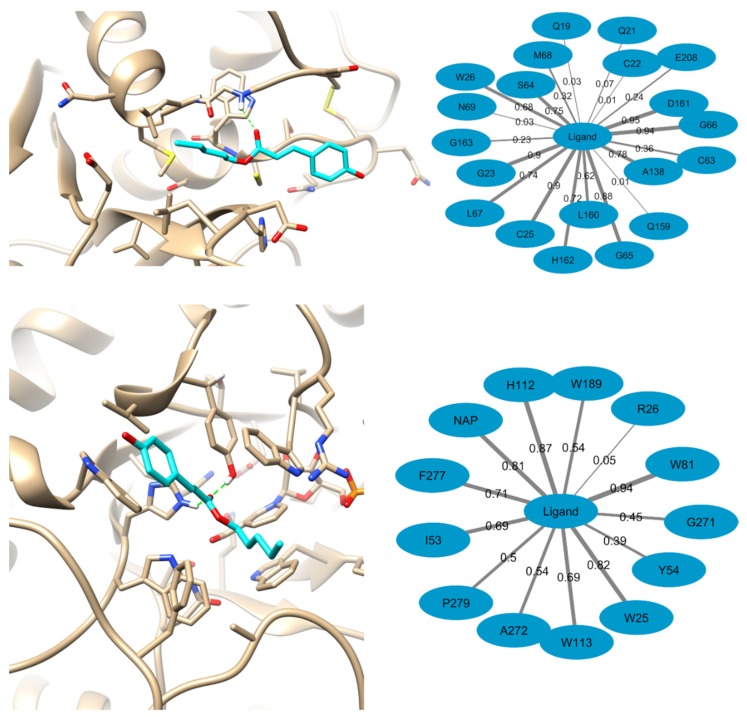
Probable binding modes of compound **7** to CZ (top) and AKR (bottom). Hydrogen bonds are represented with green lines. The predicted frequency of interaction across the 500 snapshots used for MM-PBSA calculations with the binding site residues is represented in the right. Edges width is proportional to frequency of interaction with each amino acid and the frequency values are represented in each edge.

First examined the network of interactions of compound **7** bound to AKR and CZ during the molecular dynamics simulations used for MM-PBSA calculations. The results of these analyses are represented in Figure 4. The represented structures of the complexes correspond with the representative structure of the cluster containing the largest number of molecular dynamics snapshots. Opposite to the predictions of the molecular docking results, when molecular dynamics simulations are run for the CZ-compound **7** complex, hydrogen bonds to the receptor can appear through the ligand’s carbonyl group. These hydrogen bond interactions are observed in 59% of the 500 selected simulations snapshots and they are more frequent with G66 (48% of all snapshots), followed by W26 (8%) and the catalytic C25 (3%). Also, the most frequent contacts observed (for more than 50% of the snapshots) in the complex predicted for compound **7** with CZ are with D161, G66, the catalytic C25, G23, G65, A138, S64, L67, H162, W26, and L160. These contacts are of hydrophobic and Van der Waals character. The less represented interactions appear mainly due to the flexibility of the receptor as well as because of the small size of compound **7** relative to other known CZ inhibitors which allows for its positioning in different regions of the CZ active site. Still, the fact that the ligand directly interacts with the catalytic C25 in 90% of the snapshots ensures that it is able to block the catalytic activity of the enzyme.

The predicted complex of compound **7** with AKR shows that it can be stabilized by the formation of hydrogen bonds with Y54, H112, and W113 in 57% of the selected snapshots. In addition, in 94% of them the phenol group of the ligand is observed to stack through π–π interactions with W81. The most frequent contacts of compound **7** with AKR along the MD simulations were observed with W81, H112, W25, the NAP+ cofactor, F277, W113, I53, W189, A272, and P279. The contact of the ligand with the NAP+ cofactor in 80% of the selected snapshots support the hypothesis that it can block the access of the enzyme substrates to the later and hence to inhibit the receptor’s proper functioning.

Finally, investigated the contribution of the residues in the binding pockets of AKR and CZ to gain additional insights about the binding energetics of the ligand to these to receptors. The results of this analysis in summarized in Table 4. The results indicate that for both enzymes the Van der Waals interactions between the ligand and the receptors favor its binding. In contrast, not all residues are able to make a global favorable contribution to the free energy of binding. In the predicted complex between compound **7** and AKR, W81, W25, and P279 makes the largest contribution to ligand binding. This is a consequence of the large network of hydrophobic interactions, including π-π stacking, that these residues make with the ligand’s phenol and aliphatic moieties. On the other hand, the formation of the hydrogen bonds with Y54 and H12 are associated with a high desolvation penalty that cancels free energy and contributes to these interactions.

Another example of the importance of considering the effect of desolvation is the interaction of the carbonyl group of compound **7** with G66 of CZ through hydrogen bond that is observed in 48% of the selected complex snapshots. On the other side, the interaction with W26 is highly favorable since this residue is partially buried and the van der Waals and electrostatic interactions of the ligand with it is higher that its desolvation penalty. Summarizing, the results of the free energy of binding decomposition can aid in the future optimization of the activity of this series of compounds. This can be achieved by suggesting structural modification that could improve the free energy of binding through their interaction with receptor residues which are predicted to have a low/negative impact in binding.

Given the importance of CZ and AKR in the life cycle of *T. cruzi*, the discovery of new potential inhibitors of these enzymes is crucial for the development of novel drug candidates. We consider that the results of the modeling studies performed for compound **7** strongly suggest two possible mechanisms of action for the chemical compounds herein presented. Based on these results, further experiments will be required to validate our hypothesis that this series of chemicals can serve as inhibitors of the *T. cruzi* enzymes CZ and AKR.

## 3. Conclusions

Of the twelve *p*-coumaric acid derivatives evaluated in this study, compounds **3**, **5**, **7**, and **8** presented good trypanocidal activity. Derivative **7** demonstrated the best activity against both epimastigote and trypomastigote forms, suggesting that the addition of a five-atom aliphatic carbon chain as a substituent resulted in a more potent derivative. Due to the high selectivity of compound **7**, in flow cytometry analyses we observed reductions in the percentage of viable cells, and increases in the percentage of cells labeled with 7-AAD, increases in reactive oxygen species (leading to oxidative stress on the parasite), and loss of mitochondrial membrane potentials (indicating cell death by necrosis). This mechanism was confirmed by scanning electron microscopy; the loss of cellular integrity, and cell death by necrosis being noted. Molecular docking studies confirmed the affinity of compound **7** for the CZ and AKR enzymes, which are essential for *T. cruzi* growth. The results indicate these derivatives as promising prototypes for development of new drug candidates with trypanocidal activity.

## 4. Materials and Methods

### 4.1. Chemical Characterization and Reagents

For the isolation and purification of the products, column adsorption chromatography (silica gel 60, ART 7734, MERCK, St. Louis, Missouri, EUA) on hexane and ethyl acetate gradients was used. Reaction monitoring and product analysis were via thin layer analytical chromatography (silica gel 60 F254) and ultraviolet light visualization using two wavelengths (254 and 366 nm). Infrared spectra were performed by FTIR spectrophotometry, Cary 630 FTIR Agilent Technologies^®^, potassium bromide (KBr) pills, and frequency measurement in cm^−1^. The ^1^H and ^13^C NMR spectra were obtained on a VARIAN-NMR-SYSTEM machine operating at 400 and 100 MHz. Deuterated solvent was used (DMSO-d_6_ and CDCl_3_). Chemical shifts (*d*) were measured in parts per million (ppm) with coupling constants (*J*) in Hz. High Resolution Mass spectra were performed in the Centro de Tecnologias Estratégicas do Nordeste (CETENE), Recife, PE, Brazil (www.cetene.gov.br), on Ultraflex II TOF/TOF equipment with a high performance solid state laser (λ = 355 nm) and a reflector, using MALDI technique—Matrix Assisted Laser Desorption Ionization. Samples were loaded in a steel plate (MTP 384 steel base; Bruker Daltonics Gmbh, Bremen, Germany).

### 4.2. General Procedure for Preparation of Compounds **1–8**

The *p*-coumaric acid (0.1g; 0.61mmol) was dissolved in alcohol (20 mL) in the presence of H_2_SO_4_ (0.2 mL) and refluxed till the completion of the reaction (5–27 h), and noted using single spot TLC. The solvent was evaporated under reduced pressure and water (10 mL) was added to the crude mixture. The solution was extracted with ethyl acetate (3 × 10 mL), and neutralized with 5% sodium bicarbonate (10 mL), and water (10 mL). The organic phase was dried over anhydrous sodium sulfate, and the solvent was evaporated under reduced pressure. The product was isolated on a silica gel 60 chromatographic column using hexane and ethyl acetate (7:3) as eluent [61].

### 4.3. Preparation of Compounds **9–12** by Mitsunobu Reaction

The *p*-coumaric acid (0.1g; 0.61mmol) and alcohol (0.61 mmol) were dissolved in 2.25 mL of tetrahydrofuran. The reaction mixture was stirred under magnetic stirring at 0 °C for 30 min. After addition of diisopropyl azodicarboxylate (0.12 mL; 0.61 mmol) and triphenylphosphine (0.16 g; 0.61 mmol), the reaction was performed at room temperature for 48–52 h. The solvent was removed in rotary evaporator and 10 mL of water was added to the flask. The solution was then extracted with ethyl acetate (3 × 10mL) and treated with 5% sodium bicarbonate (10 mL), and water (10 mL). The organic phase was dried over anhydrous sodium sulfate, the solvent was then evaporated under reduced pressure. The product was isolated on a silica gel 60 chromatographic column using hexane and ethyl acetate (8:2) as eluents [62].

### 4.4. Chemical Characterization of Compounds **1–12**

The data of structural analysis using IR, ^1^H and ^13^C NMR, and HRMS to compounds **1–12** are available in Supporting Information.

### 4.5. Effect of p-Coumaric Derivatives on T. cruzi Epimastigote Forms

Preparation of *T. cruzi* epimastigote was performed by cultivation in 96-well Y-staining plates at a density of 106 cells/mL, and using liver tryptose infusion with 10% fetal bovine serum (SFB) supplementation, and 1% antibiotic. They were then treated with *p*-coumaric derivatives. The grown parasites presenting inhibition were collected and quantified in a Neubauer chamber after incubation at 28 °C for 24 h [63]. To determine relative viability, parasites treated only with sterile PBS medium were used as negative controls (viability of 100%), and experiments were performed in triplicate.

### 4.6. Effect of p-Coumaric Derivatives on T. cruzi Trypomastigote Forms

Acquisition of *T. cruzi* trypomastigotes was by infection of LLCMK2 cells using trypomastigotes in T-25/75cm^2^ containers at 37 °C with 5% CO_2_ atmosphere in DMEM (Vitrocell, São Paulo, Brazil) and supplementation of 1% antibiotics and 2% SFB for 24 h. Then the trypomastigotes were plated and treated with *p*-coumaric acid derivatives. Cell viability was counted after 24 h of incubation at 37 °C. LC_50_ was obtained [64]. While relative cell viability was calculated using sterile PBS-treated cells for both negative controls and experiments, all in triplicate.

### 4.7. Cytotoxicity Evaluation (Mammalian Cells)

For cell viability we used a standard MTT assay. LLC-MK2 cells were inoculated in DMEM medium, treated with different concentrations of *p*-coumaric derivatives and incubated at 37 °C for 24 h. MTT (Amresco, Solon, Ohio, USA; 5 mg/mL) was inserted and the cells were incubated for 4 h.

In the next procedure, formazan was solubilized in 10% sodium dodecyl sulfate (SDS; Vetec, São Paulo, Brazil). For cell viability analysis, a plate reader (Biochrom^®^ Asys Expert Plus, CAMBRIGE, UK) with a wavelength of 570 nm was used. While in relative cell viability cells were calculated, which were treated with sterile PBS in triplicate for negative controls and experiments [65].

### 4.8. Statistical Analysis

Analyzes were done in triplicate. In the statistical approach we used the GraphPad Prism 5 software (GraphPad Software, San Diego, CA, USA). In the treatment of the data, one-way analysis of variance (ANOVA) and Dunnett post-test were applied. Significance was established as * *p* < 0.05.

### 4.9. Evaluation of Cell Death Mechanisms

#### 4.9.1. Principle of the Method

Evaluation of the mechanism by which compound **7** induced cell death was performed using flow cytometry with 7-AAD (7-aminoactinomicyn D) and phycoerythrin-labeled Annexin V (AxPE) as fluorescent markers. 7-AAD is a molecule capable of specifically interleaving into DNA. However, it is not able to penetrate cells with intact membranes. Cells with high 7-AAD labeling are thus considered necrotic due to loss of membrane integrity [66]. Annexin V is a 36 KDa protein that in the presence of calcium ions presents high affinity for phosphatidylserine. Thus, Ax/PE is used to evaluate external phosphatidylserine as a marker of apoptosis [67].

#### 4.9.2. Experimental Procedure

*T. cruzi* epimastigote forms were cultured at a density of 10^6^ cells/mL in the presence of compound **7** (at concentrations of 5.164 and 10.329 μM) for 24 h in a BOD greenhouse. The cells were then transferred to cytometry tubes, centrifuged at 4000 RPM for 5 min, and washed with PBS once, and then washed twice with binding buffer (pH 7.4; 10 mM HEPES solution; 140 mM NaCl and 2.5 CaCl_2_ mM). After final centrifugation, the pellet was re-suspended in 100 µl binding buffer, 5 µl 7-AAD (0.5 mg/mL), and 5 µl Ax/PE. The cell death mechanism procedure was performed using a commercial kit (Annexin V PE Apoptosis Detection Kit I, BD Biosciences, San José, Califórnia, EUA) following the manufacturer’s guidelines. After 15 min of incubation in the dark, 400 µl of binding buffer was added to each tube and cells were analyzed on FACSCalibur (BD Biosciences, San José, Califórnia, EUA).

For each tube, a minimum of 10^4^ cells were analyzed for quantitation of the percentage of unlabeled, and individually or double labeled 7-AAD and Ax/PE cells. For this, an argon laser (488 nm) was used to excite the fluorochromes. Ax/PE labeled cells were read by an FL2 detector (563 at 606 nm, yellow fluorescence), while 7-AAD labeled cells were read at 615 to 645 nm wavelength (red fluorescence, FL3 detector).

### 4.10. Analysis of Cytoplasmic Reactive Oxygen Species

#### 4.10.1. Principle of the Method

The increased presence of reactive oxygen species (ROS) in the cytoplasm of compound **7** treated epimastigote cells was analyzed using the 2′,7′-dichlorofluorescein diacetate reagent (DCFH-DA). As a lipophilic molecule, when internalized in cells it is cleaved by cytoplasmic esterase into DCFH (reduced 2′-7′-dichlorofluorescein), and as it is unable to cross membranes DCFH reacts with intra-cytoplasmic ROSs producing DCF (oxidized 2′-7′-dichlorofluorescein). DCF emits green fluorescence, and the fluorescent intensity emitted by the cell is directly proportional to the intra-cytoplasmic ROS concentration [68].

#### 4.10.2. Experimental Procedure

A concentration of 10^6^ epimastigotes/mL were placed in 24-well plates in the presence of BIS (5.164 and 10.329 μM). After 3 h of incubation, 10 µL of a DCFH-DA solution (2 mM in DMSO) was added to give a final concentration of 20 µM. After 24 h of incubation with compound **7**, the cells were harvested, centrifuged (2800 RPM for 7 min), washed with PBS twice, and re-suspended in 500 µL PBS. At the end, the cells were analyzed by flow cytometer.

In accordance with Kessler et al. (2013) [69], the level of labeling in each group was assessed by the mean geometric fluorescence intensity. The results obtained were expressed as relative fluorescence intensity using the formula:

(1) $$Relative\;fluorescence=\frac{mTEST}{mCONTROL}$$

where:

*mTEST* = geometric mean of the group to be analyzed;
*mCONTROL* = geometric mean of the control group.

### 4.11. Assessment of Mitochondrial Transmembrane Potential

#### 4.11.1. Principle of the Method

Mitochondrial transmembrane potential (ΔΨm) was assessed using rhodamine 123 fluorescent dye (Rho123) [70,71].

**Figure 5:**
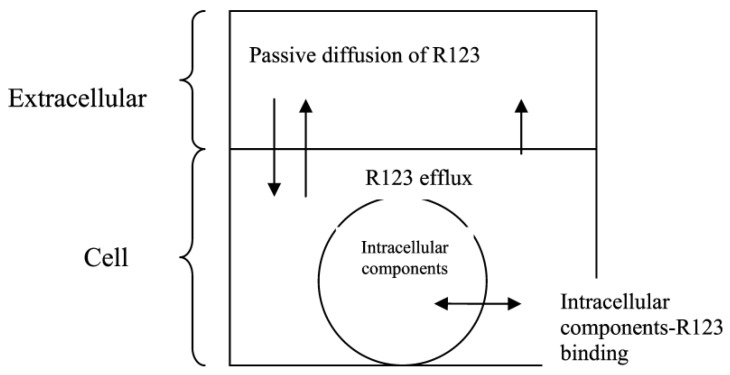
Mitochondrial labeling mechanism using rhodamine 123 (Rho123).

Rhodamine 123 (Rho123) is a lipophilic and cationic dye that under normal conditions accumulates in the mitochondrial inter-membrane space. Presenting affinity with hydrogen ions, there is a reduction in the concentration of H^+^ ions in the inter-membrane space, which decreases Rho123 accumulation, as illustrated in Figure 5. Changes in ΔΨm can be measured using Rho123, measuring the decrease in green fluorescence [72].

#### 4.11.2. Experimental Procedure

The compound **7** (5.164 and 10.329 μM) was added to the epimastigotes (10^6^ cells) in 24-well plates. At 24 h of incubation, cells were transferred to tubes, centrifuged and washed with PBS. The cells were then re-suspended in 100 µL PBS, and 5 µL of Rho123 solution (200 µg/mL in absolute ethanol) was added to obtain a final concentration of 10 µg/mL. Samples were incubated in the dark for 30 min, washed twice with PBS, and re-suspended in PBS (500 µL/tube). Finally, the samples were analyzed using flow cytometry [73]. Results were evaluated using relative fluorescent intensity, calculated as described in the previous item.

### 4.12. Scanning Electron Microscopy

#### Evaluation of Morphological Changes in *T. cruzi* Induced by Compound **7**

For evaluation of changes in *T. cruzi* ultrastructure induced by compound **7**, the epimastigote forms were incubated together with compound **7** for 24 h. After this period, the samples were centrifuged (5000× rpm; 10 min), the supernatant discarded, and the pellet re-suspended in glutaraldehyde solution (2.5%) and incubated for 2 h. The fixed samples were again centrifuged and exposed to increasing concentrations of ethanol (30%, 50%, 70%, 90%, and 100%) for dehydration, followed by centrifugation (5000× rpm; 5 min). Finally, the cells were transferred to the surface of circular coverslips (15 mm) and dried in a CO_2_ oven. The coverslips were covered with a gold layer (20 nm thickness) using the QT150 ES-Quorum Metalizer and analyzed using a Quanta 450 FEG-FEI Scanning Electron Microscope for observation of changes in the three-dimensional structure of the epimastigote forms of *T. cruzi*.

### 4.13. Computational Methods

#### 4.13.1. General Modeling Workflow

The first step of the computational modeling workflow was to identify potential *T. cruzi* targets for compound **7** employing a consensus target fishing strategy. A second set of well-known anti-trypanosomal targets were also added to the previous identified ones. The compound was docked to each potential target its possible binding modes. The free energies of binding of compound 7 to its potential targets were estimated using MM-PBSA calculations. The free energy calculations were performed from molecular dynamics (MD) simulations of the predicted complexes. Only the parameters of the simulations that changed from their default values are listed in the below sections. UCSF Chimera was used to depict molecular structures [74] and network interactions were visualized with Cytoscape [75].

#### 4.13.2. Targets Selection

The most potent compound of the series (compound **7**) was used for the identification of potential molecular targets using three different computational target fishing approaches: MolTarPred [76], PredictionCharite [77], and SEA [78]. The appearance in the lists of predicted targets provided by all the three above listed methods was used as consensus criterion for the selection of potential targets for compound **7**.

Computational target fishing is based on the similarity principle and the reference data for the similarity search is usually extracted from databases such as ChEMBL [79] and PubChem [80]. These databases are biased against ligand-target interactions for human proteins, thus predicted targets will suffer the same bias. Taking this bias into account, non *T. cruzi* predicted targets were used as query for Blast [81] searches against the *T. cruzi* genome (taxid:5693) for the identification of possible orthologous proteins in the parasite. In addition, known targets of anti-trypanosomal compounds were also considered for further modeling.

#### 4.13.3. Molecular Docking

The structures of the *T. cruzi* aldo-keto reductase (PDB 4GIE), cruzain (PDB 1U9Q), glyceraldehyde-3-phosphate dehydrogenase (PDB 1QXS), mitochondrial iron-dependent superoxide dismutase (PDB 4DVH), cytoplasmatic iron-dependent superoxide dismutase (PDB 2GPC) and trypanothione reductase (PDB 4NEW) were obtained from the Protein Data Bank [82]. For the *T. cruzi* carbonic anhydrase, the homology model deposited at the Swiss-Model server [83] (https://swissmodel.expasy.org/repository/uniprot/A0A2V2XG02) was employed.

The most sTable 3D conformation of compound **7** was obtained by means of the OMEGA software and AM1-BCC charges were added to the ligands with MOLCHARGE [84]. The Gold software [85] was used for molecular docking calculations. All water and ligand molecules were removed from targets for docking. The binding sites of the receptors were defined as a region within 10 Å of a binding pocket atom or as the region within 6 Å from the bound reference ligands. Gold was run for 30 different initial populations using CHEMPLP as the primary scoring function. The Search efficiency parameter of Gold was set to 200% (Very Flexible). The 30 ligand-receptor complexes predicted for each target were rescored using the GoldScore, ChemScore, and ASP scoring functions.

To select the best conformer of the ligands, a consensus scoring approach was implemented. For this, the score of conformer *Ci* according to the scoring function *Sj* (*S_i_*_,*j*_) was scaled as:

(2) $$Z_{i,j}=\frac{S_{i,j}-\bar{S_{j}}}{std\left(S_{j}\right)}\;(\mathrm{Equation}\;(1))$$

where $S_{j}$ is the mean of the scoring function *S_j_* across all conformers and std (*S_j_*) is the standard deviation of the *S_j_* values. Next, the scaled scores of each conformer *C_i_* were averaged to obtain the consensus score *Z_i_*. The most probable binding modes of the compound **7** were selected as those with *Z_i_* > 0.

#### 4.13.4. Molecular Dynamics Simulations and MM-PBSA Calculations

Molecular dynamics simulations were performed with Amber 2018 [86] using the ff14SB and gaff force fields for proteins and non-aminoacidic residues, respectively. All probable complexes of compound **7** with the studied targets were subject to the same modeling procedure. All systems were modeled in explicit solvent following the procedure described below.

Compound **7** topology and the corresponding forcefield modifications with generated with antechamber. A truncated octahedron box extending 10 Å from complex was constructed. The system was solvated with TIP3P water molecules and excess charges were neutralized by the addiction of either Na^+^ or Cl^–^ ions. Afterward, the system was minimized for 500 steps of the steepest descent method followed by 500 cycles of conjugate gradient at constant volume with all atoms except water molecules restrained using a force constant of 500 kcal/mol.Å2. Long range electrostatic interactions were treated according to the PME method with a cutoff of 12 Å. Next, a second minimization stage was carried out with no restrains with a PME cutoff of 10 Å for 1500 steps of steepest descent method followed by 1000 cycles of conjugate gradient at constant volume.

Energy minimization was followed by system heating from 0 to 300 K at constant volume using a 10 Å cutoff for PME and with all atoms except water molecules restrained with a force constant of 10 kcal/mol.Å2. Bonds involving hydrogens were constrained using the SHAKE algorithm and their interactions were omitted. A Langevin thermostat with a collision frequency of 1.0 ps^−1^ was employed. Heating was performed for 10,000 steps with a time step of 2 fs.

After heating, the system was equilibrated for 100 ps at 300 K and at constant pressure of 1 bar using isotropic position scaling with a relaxation time of 2 ps. The cutoff distance for PME was set to 10 Å. Bonds involving hydrogens were constrained using the SHAKE algorithm and their interactions were omitted. Temperature was controlled employing a Langevin thermostat with a collision frequency of 1.0 ps^−1^. The equilibrated system was used as input for 50 different molecular dynamics simulations of 2 ns length. Each of these simulations were performed with different initial velocities.

MM-PBSA calculations to estimate the free binding energy of the complexes were performed with AmberTools 18 [86]. For this, 500 snapshots (one every 200 ps) spanning the 50 previously obtained molecular dynamics trajectories were employed for each target-compound **7** complex. The ionic strength was set to 100 mM for all systems. Default implicit solvent parameters were set for MM-PBSA calculations.

## Figures and Tables

**Table 1:** Trypanocidal activity of compounds **1**–**12** against Y strain of *T. cruzi* and LLC-MK2.

*p*-Coumaric Esters					
	Y strain		Y strain		
	(Epimastigotes)		(Tripomastigotes)		LLC-MK2
	IC_50_ (μM)	SI	IC_50_ (μM)	SI	IC_50_ (μM)
**1**	601.06 ± 249.17				>561.21
**2**	216.06 ± 109.25				443.41 ± 109.25
**3**	16.76 ± 1.93	26.55	117.09 ± 59.15	3.8	445.55 ± 187.16
**4**	334.90 ± 54.30				289.71 ± 48.97
**5**	32.03 ± 5.39	>14.05	360.42 ± 112.49	>1.25	>449.96
**6**	154.99 ± 33.14				273.89 ± 46.76
**7**	5.16 ± 1.28	23.86	61.63 ± 28.59	2	123.26 ± 22.62
**8**	13.23 ± 2.56	9.1	111.31 ± 38.70	1.08	120.32 ± 19.20
**9**	68.86 ± 18.12				131.32 ± 16.91
**10**	50.55 ± 18.94				141.39 ± 15.18
**11**	75.47 ± 13.41				160.18 ± 97.64
**12**	40.05 ± 10.12				2.74 ± 9.11

**Table 2:** Results of the molecular docking of compound **7** to its potential molecular targets.

Target	Conformer	CHEMPLP		GoldScore		ChemScore		ASP		Consensus Z-Score
		Score	Z-Score	Score	Z-Score	Score	Z-Score	Score	Z-Score	
CA	1	49.10	2.02	20.96	1.55	19.49	1.33	21.82	−0.24	1.17
AKR	1	56.39	2.66	29.48	2.02	23.39	1.07	29.91	−0.64	1.28
	2	52.95	1.25	29.35	1.98	21.63	−0.19	35.77	1.98	1.25
	3	53.55	1.50	23.25	0.13	23.85	1.40	34.20	1.28	1.08
CZ	1	48.74	2.44	25.33	1.60	12.71	0.21	16.45	0.83	1.27
	2	44.60	0.58	20.03	−0.23	14.08	1.18	20.10	2.55	1.02
SOD-A	1	40.44	1.43	22.70	1.56	14.33	1.24	25.08	1.61	1.46
	2	39.44	1.01	19.10	0.50	15.20	1.88	25.10	1.62	1.25
SOD-B	1	45.11	2.83	13.65	0.27	14.59	0.80	22.45	0.54	1.11
TR	1	48.30	1.53	24.73	1.31	17.34	1.76	23.78	1.25	1.46
	2	45.41	0.57	24.56	1.26	15.73	0.25	26.48	2.44	1.13
GAPDH ^(a)^	1	42.90	2.35	20.61	1.36	13.26	1.80	13.79	−0.01	1.37
	2	40.97	1.56	21.16	1.56	13.01	1.60	14.72	0.26	1.24
	3	41.83	1.92	18.52	0.59	12.89	1.50	14.47	0.19	1.05
GAPDH+NAD ^(b)^	1	44.37	2.47	23.51	0.99	13.25	1.34	15.69	0.53	1.33

^(a)^ GAPDH with bound cofactor. ^(b)^ GAPDH without bound cofactor. CA: caffeic acid; AKR: aldo-keto reductases; SOD-A: mitochondrial iron-dependent superoxide dismutase; SOD-B: cytoplasmatic iron-dependent superoxide dismutase; TR: trypanothione reductase.

**Table 3:** Summary of the MM-PBSA calculations for the estimation of the free energy of binding of compound **7** to its potential targets. ΔG is expressed in kcal/mol.

Target	Conformer	MM-PBSA Component							ΔG Total
		Vdwaals	EEL	EPB	ENPOLAR	EDISPER	ΔG Gas	ΔG Solv	
CA	1	−21.95	−8.52	20.85	−19.61	32.47	−30.47	33.71	3.24
AKR	1	−18.68	−3.84	13.79	−15.23	25.38	−22.52	23.95	1.43
	2	−22.98	−13.56	24.72	−19.13	31.65	−36.54	37.24	0.70
	3	−25.04	−8.37	17.98	−20.41	32.43	−33.41	30.00	−3.42
CZ	1	−28.47	−4.95	15.34	−21.01	34.94	−33.42	29.27	−4.15
	2	−26.87	−4.96	21.43	−19.48	32.47	−31.83	34.43	2.60
SOD-A	1	−34.61	−14.76	35.23	−25.32	42.74	−49.37	52.64	3.27
	2	−33.36	−13.14	33.30	−24.53	42.19	−46.51	50.96	4.45
SOD-B	1	−32.10	−22.17	47.67	−25.49	43.36	−54.27	65.54	11.27
TR	1	−24.51	−9.66	21.91	−20.20	32.74	−34.17	34.45	0.28
	2	−22.75	−3.72	15.00	−18.36	30.81	−26.47	27.45	0.97

**Table 4:** Energy decomposition of the free energy of binding across residues in the binding pockets of AKR and CZ. Energy is expressed in kcal/mol.

CZ					AKR				
Residue	Van der Waals	Electrostatic	Polar Solvation	ΔG TOTAL	Residue	Van der Waals	Electrostatic	Polar Solvation	ΔG TOTAL
W26	−0.98	−0.65	0.52	−1.11	W81	−1.45	−0.28	0.34	−1.39
G65	−0.76	−1.33	1.17	−0.92	W25	−1.84	0.33	0.24	−1.28
H162	−1.39	−0.27	0.84	−0.82	P279	−0.97	0.09	−0.11	−1.00
L160	−0.97	0.10	0.25	−0.62	F277	−1.31	0.08	0.26	−0.97
A138	−0.45	0.06	−0.15	−0.54	W189	−0.74	−0.11	0.01	−0.84
L67	−0.79	0.24	0.03	−0.52	I53	−0.95	−0.25	0.50	−0.70
C25	−0.76	−0.05	0.31	−0.50	W113	−0.97	−0.97	1.54	−0.41
M68	−0.54	0.03	0.09	−0.42	A272	−0.46	0.03	0.03	−0.41
G23	−0.97	0.16	0.40	−0.42	H112	−0.51	−1.70	1.97	−0.24
G163	−0.48	0.06	0.05	−0.37	Y54	−0.36	−0.76	1.20	0.08
S64	−0.90	−0.16	0.75	−0.32	R26	−0.21	0.27	0.05	0.11
C22	−0.23	−0.11	0.06	−0.28	G271	−0.44	0.05	0.77	0.38
E208	−0.36	−0.17	0.39	−0.14					
Q159	−0.11	−0.04	0.06	−0.09					
D161	−1.55	−0.54	2.06	−0.03					
N69	−0.04	0.00	0.06	0.01					
Q21	−0.12	−0.05	0.19	0.01					
C63	−0.39	−0.15	0.62	0.08					
Q19	−0.17	0.21	0.15	0.19					
G66	−0.80	−0.46	2.02	0.76					

## Supplementary Materials

Supplementary materials can be found at https://www.mdpi.com/1422-0067/20/23/5916/s1.
