# Supplementary material for: Trypanocidal Mechanism of Action and in silico Studies of p-Coumaric Acid Derivatives
Source: Int J Mol Sci. 2019 Nov 25;20(23):5916. doi: 10.3390/ijms20235916 (PMC6928761; doi:10.3390/ijms20235916)
Supplement: Supplementary file 1 [file ijms-20-05916-s001.pdf]

Supporting Information

Trypanocidal Mechanism of Action and *in silico* Studies of *p*-Coumaric Acid Derivatives

Susiany P. Lopes[1], Yunierkis P. Castillo [2], Marilia L. Monteiro[3], Ramon R. P. P de Menezes[3], Reinaldo N. Almeida[4], Alice M. C. Martins[3], and Damião P. de Sousa[1,5*]

[1] PostGraduation Program in Technological Development and Innovation in Medicines, Federal University of Paraíba, CEP 58051-970, João Pessoa, Paraíba, Brazil; susiany_lopes@hotmail.com

[2] Escuela de Ciencias Físicas y Matemáticas, Universidad de Las Américas, Quito, 170125, Ecuador; yunierkis@gmail.com

[3] Department of Clinical and Toxicological Analysis, Federal University of Ceará, CEP 60020-181, Fortaleza, Ceará, Brazil; marilialopesmonteiro@yahoo.com.br, ramonppessoa@ufc.br, martinsalice@gmail.com

[4] Postgraduate Program in Natural and Synthetic Bioactive Products, Federal University of Paraíba, CEP 58051-970, João Pessoa, Paraíba, Brazil; reinaldoan@uol.com.br

[5] Department of Pharmaceutical Sciences, Federal University of Paraíba, CEP 58051-970, João Pessoa, Paraíba, Brazil.; damiao_desousa@yahoo.com.br

* Correspondence: damiao_desousa@yahoo.com.br

Abstract: *Trypanosoma* species are responsible for chronic and systemic infections in millions of people around the world, compromising life quality, and family and government budgets. This group of diseases is classified as neglected and causes thousands of deaths each year. In the present study, the trypanocidal effect of a set of 12 ester derivatives of the *p*-coumaric acid was tested. Of the test derivatives, pentyl *p*-coumarate (**7**) (5.16 ± 1.28 μM; 61.63 ± 28.59 μM) presented the best respective trypanocidal activities against both epimastigote and trypomastigote forms. Flow cytometry analysis revealed an increase in the percentage of 7-AAD labeled cells, an increase in reactive oxygen species, and a loss of mitochondrial membrane potential; indicating cell death by necrosis. This mechanism was confirmed by scanning electron microscopy, noting the loss of cellular integrity. Molecular docking data indicated that of the chemical compounds tested, compound **7** potentially acts through two mechanisms of action, whether by links with aldo-keto reductases (AKR) or by comprising cruzain (CZ) which is one of the key *Trypanosoma cruzi* development enzymes. The results indicate that for both enzymes, Van der Waals interactions between ligand and receptors favor binding and hydrophobic interactions with the phenolic and aliphatic parts of the ligand. The study demonstrates that *p*-coumarate derivatives are promising molecules for developing new prototypes with antiprotozoal activity.

Keywords: Natural products; esters; cinnamic acid; phenylpropanoid; *Trypanosoma*; Neglected diseases; antiparasitic activity

---

1. Experimental

1.1. Chemical characterization *compounds* ***1-12***

(*E*)-Methyl 3-(4-hydroxyphenyl)acrylate (**1**): white solid; Yield 87.98% (95.5 mg, 0.53 mmol); m.p.: 135-136 °C (lit. 129-132 °C, [2]); TLC (7:3 hexane/EtOAc), R_f= 0.52; IR υmax (KBr, cm^{-1}): 3379, 3051, 3021, 2954, 1737, 1634, 1601, 1434, 1284, 1172; ^1H NMR (DMSO-d_6, 400 MHz): δH 3.69 (3H;

s), 6.38 (1H; *d*; *J* =16 Hz), 6.81-6.79 (1H; *d*; *J*=8.60 Hz), 7.55-7.53 (1H; *d*; *J*=8.44 Hz), 7.58 (1H; *s*); ^{13}C NMR (DMSO-d_6, 100 MHz): δC 51.3, 114.0, 115.9, .125.2, 130.4, 144.9, 159.8, 167.1 [1, 2].

(*E*)-Ethyl 3-(4-hydroxyphenyl)acrylate (**2**): white solid; Yield 84.21% (295.8 mg, 1.53 mmol); m.p.: 76-77 °C (lit. 72-73 °C, [3]); TLC (7:3 hexane/EtOAc), R_f = 0.55; IR υmax (KBr, cm^{-1}): 3286, 3069, 3026, 2985, 1746, 1634, 1604, 1440, 1280, 1169; ^1H NMR ($CDCl_3$, 400 MHz): δH 1.33 (3H; *t*; *J*=7.16 Hz), 4.26 (2H; *quart*, *J*=7.16Hz), 6.28 (1H; *d*; *J*=15.94 Hz), 6.88-6.86 (2H; *d*; *J*=8.64 Hz); 7.40-7.38 (2H, *d*, *J*=8.64 Hz); 7.63 (1H; *d*; *J*=15.96 Hz); ^{13}C NMR ($CDCl_3$, 100 MHz): δC 14.4, 61, 115.0, 116.1, 126.8, 130.2, 145.5, 158.7, 168.5 [1, 3, 4].

(*E*)-Propyl 3-(4-hydroxyphenyl)acrylate (**3**): White solid; Yield 58.43% (220 mg, 1.06 mmol); m.p.: 74-75 °C (lit. 148-150 °C, [1]); TLC (7:3 hexane/EtOAc), R_f = 0.57; IR υmax (KBr, cm^{-1}): 3376, 3060, 3028, 2970, 1737, 1638, 1603, 1441, 1271, 1171; ^1H NMR ($CDCl_3$, 400 MHz): δH 0.99 (3H; *t*; *J*=7.4 Hz), 1.73 (2H; *sext*; *J*=7.44), 4.16 (2H; *t*; *J*=6.72 Hz), 6.30 (1H; *d*; *J*=15.95 Hz); 6.86-6.84 (2H, *d*, *J*=8.66 Hz); 7.42- 7.40 (2H; *d*; *J*=8.67 Hz), 7.63(1H, *d*, *J*=15.96 Hz); ^{13}C NMR ($CDCl_3$, 100 MHz): δC 10.8, 22.2, 66.5, 115.4, 116.0, 127.1, 130.3, 145.0, 158.3, 168.5 [1].

(*E*)-Isopropyl 3-(4-hydroxyphenyl)acrylate (**4**): White solid; Yield 60.73% (228.9 mg, 1.10 mmnol); m.p.: 71-72 °C (lit. 71-73 °C, [1]); TLC (7:3 hexane/EtOAc), R_f = 0.57; IR υmax (KBr, cm^{-1}): 3272, 3068, 3028, 2939, 1739, 1629, 1605, 1466, 1279, 1172; ^1H NMR ($CDCl_3$, 400 MHz): δH 1.31 (6H; *d*; *J*=6.28 Hz), 5.14 (1H; *hept*; *J*=6.25Hz), 6.27 (1H; *d*; *J*=15.94 Hz); 6.88-6.86 (2H, *d*, *J*=8.60 Hz); 7.40-7.37 (2H; *d*; *J*=8.67 Hz), 7.61(1H, *d*, *J*=15.95 Hz); ^{13}C NMR ($CDCl_3$, 100 MHz): δC 22.2, 68.4, 115.6, 116.1, 126.8, 130.3, 145.0, 158.7, 168.1 [1].

(*E*)-2-methoxyethyl 3-(4-hydroxyphenyl)acrylate (**5**): white solid; Yield 42.76% (173.7 mg, 0.78 mmol) ; m.p.: 96-97 °C; TLC (7:3 hexane/EtOAc), R_f = 0.33; IR υmax (KBr, cm^{-1}): 3260, 3062, 3040, 2959, 1735, 1655, 1609, 1447, 1266, 1176; ^1H NMR ($CDCl_3$, 400 MHz): δH 3.46 (3H; *s*), 3.71 (2H; *t*, *J*=4.64 Hz), 4.37 (2H, *t*, *J*=4.52 Hz), 6.21 (1H, *d*, *J* =15.94 Hz), 6.83-6.80 (2H, *d*; *J*=8.55 Hz), 7.35-7.32 (2H, *d*, *J*=8.7 Hz), 7.60 (1H, *d*, *J* =15.95 Hz) ^{13}C NMR ($CDCl_3$, 100 MHz): δC 58.8, 63.2, 70.8 114.6, 116.1, 126.5, 130.1, 145.6, 158.8, 167.8 [1].

(*E*)-Butyl 3-(4-hydroxyphenyl)acrylate (**6**): Brown amorphous; Yield 98% (395.5 mg, 1.79 mmol) ; TLC (7:3 hexane/EtOAc), R_f = 0.65; IR υmax (KBr, cm^{-1}): 3277, 3062, 3028, 2959, 1737, 1634, 1604, 1474, 1280, 1172; ^1H NMR ($CDCl_3$, 400 MHz): δH 0.96 (3H; *t*; *J*=7.38 Hz), 1.44 (2H; *sext*; *J*=7.44), 1.69 (2H; *pent*; *J*=6.80 Hz), 4.20 (3H; *t*, *J*=6.67); 6.30 (1H; *d*; *J*=15.94 Hz); 6.85-6.83 (2H, *d*, *J*=8.59 Hz); 7.43-7.41 (2H; *d*; *J*=8.59 Hz), 7.62 (1H, *d*, *J*=15.95 Hz); ^{13}C NMR ($CDCl_3$, 100 MHz): δC 14.0, 19.3, 30.8, 64.8, 115.2, 116.1, 126.8, 130.2, 145.2, 158.5, 168.5 [1].

(*E*)-Pentyl 3-(4-hydroxyphenyl)acrylate (**7**): Brown amorphous; Yield 44.09% (188.8 mg, 0.80 mmol) ; TLC (7:3 hexane/EtOAc), R_f = 0.60; IR υmax (KBr, cm^{-1}): 3400, 3069, 3014, 2959, 1735, 1633, 1605, 1445, 1268, 1170; 1H NMR ($CDCl_3$, 400 MHz): δH 0.94 (3H, *t*, *J*=6.99), 1.40 (2H, *m*), 1.40 (2H, *m*), 1.73 (2H, *quint*, *J*=7.32 Hz), 4.22 (2H, *t*, *J*=6.74 Hz), 6.32 (1H, *d*, *J*=15.95 Hz), 6.90-6.87 (2H, *d*; *J*=8.62 Hz), 7.43-7.41 (2H, *d*, *J*=8.62 Hz), 7.65 (1H, *d*, *J*=15.94 Hz) ^{13}C NMR ($CDCl_3$, 100 MHz): δC 14.1, 22.5, 28.2, 28.5, 65.1, 115.2, 116.1, 126.9, 130.2, 145.1, 158.5, 168.4 [1].

(*E*)-Isopentyl 3-(4-hydroxyphenyl)acrylate (**8**): Brown solid; Yield 79.36% (339.8 mg, 1.45 mmol); m.p.: 111-112 °C (lit. 151-153 °C, [1]); TLC (7:3 hexane/EtOAc), R_f = 0.66; IR υmax (KBr, cm^{-1}): 3380, 3071, 3028, 2958, 1741, 1636, 1604, 1437, 1277, 1167; ^1H NMR ($CDCl_3$, 400 MHz): δH 0.95 (3H, *d*, *J*=6.64 Hz), 1.72 (1H, *quart*, *J*=6,75 Hz), 1.60 (2H, *d*, *J*= 6,74 Hz), 4.23 (2H, *t*, *J*=6.83 Hz), 6.31 (1H, *d*, *J*=15.96 Hz,), 6.87-6.85 (2H, *d*; *J*=8.44 Hz), 7.40-7.38 (2H, *d*, *J*=8.49 Hz), 7.64 (1H, *d*, *J*=15.98 Hz) ^{13}C NMR ($CDCl_3$, 100 MHz): δC 22.6, 25.3, 37.5, 63.6, 115.2, 116.1, 126.8, 130.2, 145.1, 158.6, 168.6 [1].

(*E*)-Hexyl 3-(4-hydroxyphenyl)acrylate (**9**): White solid; Yield 36.88% (167.4 mg; 0.67 mmol); m.p.: 42-43 °C (lit. 160 °C, [6]); TLC (8:2 hexane/EtOAc), R_f = 0.47; IR υmax (KBr, cm^{-1}): 3383, 3058,

3028, 2955, 1737, 1625, 1604, 1472, 1276, 1170; ^{1}H NMR ($CDCl_3$, 400 MHz): δH 0.89 (3H, *t*, *J*=6.02), 1.36 (6H, *m*), 1.69 (2H, *quint*; *J*=16.0 Hz), 4.19 (2H, *t*, *J*=6.74 Hz), 6.28 (1H, *d*, *J*=15.94 Hz,), 6.88-6.86 (2H, *d*; *J*=8.64 Hz), 7.42-7.40 (2H, *d*, *J*=8.62 Hz), 7.63 (1H, *d*, *J*=15.95 Hz) ^{13}C NMR ($CDCl_3$, 100 MHz): δC 14.1, 22.7, 25.8, 28.8, 31.6, 65.1, 115.4, 116.1, 127, 130.1, 145.0, 158.4, 168.3 [4, 6].

(*E*)-Dodecyl 3-(4-hydroxyphenyl)acrylate (**10**): White solid; Yield 29.55% (179.6 mg, 0.54 mmol); m.p.: 76-77 °C (lit. 73-75 °C, [5]); TLC (8:2 hexane/EtOAc), R_f = 0.52; IR υmax (KBr, cm^{-1}): 3381, 3073, 3032, 2926, 1731, 1625, 1603, 1473, 1275, 1171; ^{1}H NMR ($CDCl_3$, 400 MHz): δH 0.88 (3H, *t*, *J*=6.68),1.35 (18H, *m*), 1.70 (2H, *quint*, *J* = 6.72 Hz), 4.20 (2H, *t*, *J* = 6.76 Hz), 6.30 (1H, *d*, *J*=15.96 Hz,), 6.88-6.86 (2H, *d*; *J*=8.64 Hz), 7.42-7.40 (2H, *d*, *J*=8.64 Hz), 7.63 (1H, *d*, *J*=15.96 Hz) ^{13}C NMR ($CDCl_3$, 100 MHz): δC 14.1, 22.7, 26, 28.7, 29.3, 29.4, 29.5, 29.6, 29.6, 29.7, 31.9, 64.9, 115.3, 116, 126.9, 130, 145.1, 158.5, 168.5 [5].

(*E*)-4-Methylbenzyl 3-(4-hydroxyphenyl)acrylate (**11**): White solid; Yield 30% (147.2 mg, 0.54 mmol); m.p.: 93-94 °C (lit. 106-108 °C, [7]); TLC (8:2 hexane/EtOAc), R_f = 0.34; IR υmax (KBr, cm^{-1}): 3291, 3066, 3027, 2958, 1737, 1629, 1605, 1450, 1279, 1164; ^{1}H NMR ($CDCl_3$, 400 MHz): δH 2.37(3H, *s*), 5.22 (2H, *s*), 6.33 (1H, *d*, *J*= 15.94 Hz), 6.86-6.84 (2H, *d*; *J*=8.76 Hz), 7.20-7.18 (2H, *d*, *J*=7.8 Hz), 7.32-7.30 (2H, *d*, *J*=8.04 Hz), 7.40-7.38 (2H, *d*, *J*=8.72 Hz), 7.66 (1H, *d*, *J*=15.96 Hz) ^{13}C NMR ($CDCl_3$, 100 MHz): δC 21.7, 66.6, 115.1, 116.1, 127.0, 128.6, 129.4, 130.2, 133.1,138.3, 145.5, 158.3, 168.0 [7].

(*E*)-4-Isopropylbenzyl 3-(4-hydroxyphenyl)acrylate (**12**): White solid; Yield 29.76% (161.2 mg, 0.54 mmol); m.p.: 107-108 °C; TLC (8:2 hexane/EtOAc), R_f = 0.39; IR υmax (KBr, cm^{-1}): 3232, 3056, 3031, 2960, 1737, 1638, 1605, 1455, 1226, 1158; ^{1}H NMR ($CDCl_3$, 400 MHz): δH 1,27 (6H, *d*,*J*=6.92 Hz), 2.94 (1H, *sept*, *J*= 6,74Hz), 5.24 (2H, *s*), 6.35 (1H, *d*, *J*=15.94 Hz), 6.87-6.85 (2H, *d*; *J*=8.55 Hz),7.28 (2H,*m*), 7.38-7.36 (2H,*d*, *J*=8.2), 7.43-7.41 (2H, *d*, *J*=8.4 Hz), 7.69 (1H, *d*, *J*=15.96 Hz) ^{13}C NMR ($CDCl_3$, 100 MHz): δC 24.1, 34.2, 66.6, 128.6, 126.8, 149.3, 126.8, 128.6, 133.5, 115.2, 116.1, 127.1, 130.2, 145.3, 158.2, 167.9 [7]. HRMS (MALDI) calculated for $C_{19}H_{20}O_3$ $[M + Na]^+$: 319.1309, found 319.1332.

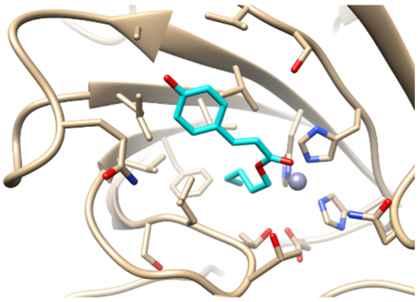

***FS 1**. Predicted binding mode of compound **7** to CA.*

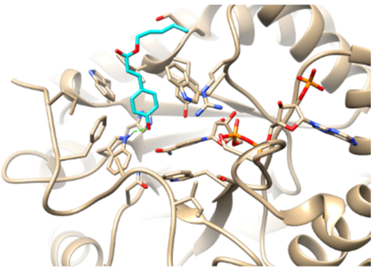

***FS 2** Predicted binding mode of compound **7** to AKR. Conformer 1*

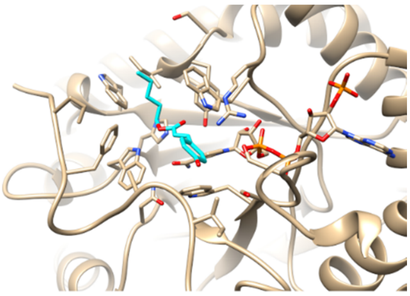

FS 3 *Predicted binding mode of compound* ***7*** *to AKR. Conformer 2*

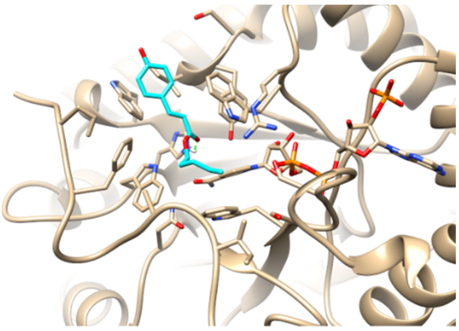

FS 4 *Predicted binding mode of compound* ***7*** *to AKR. Conformer 3*

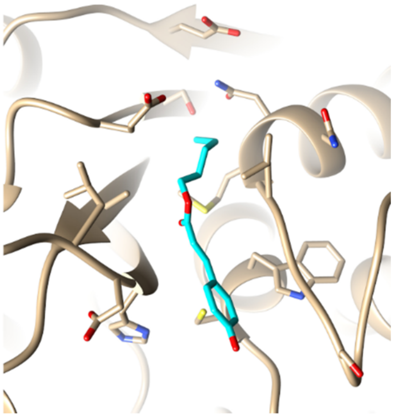

FS 5 *Predicted binding mode of compound* ***7*** *to CZ. Conformer 1*

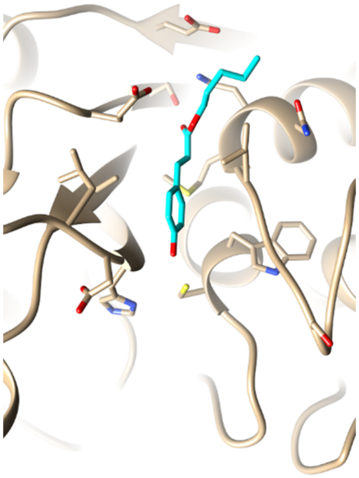

FS 6 *Predicted binding mode of compound* ***7*** *to CZ. Conformer 2*

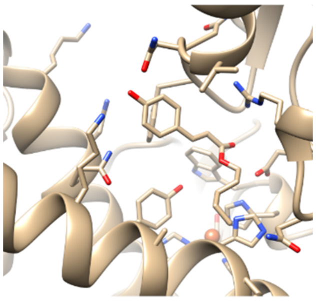

FS 7 *Predicted binding mode of compound* ***7*** *to SOD-A. Conformer 1*

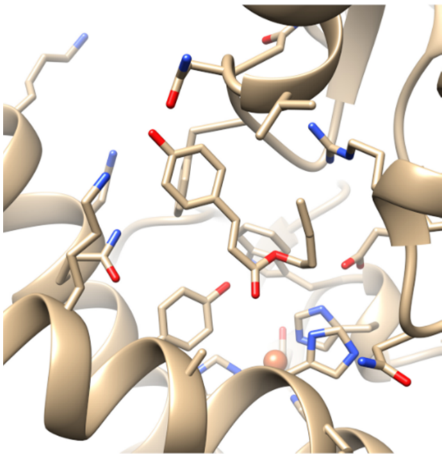

FS 8 *Predicted binding mode of compound* ***7*** *to SOD-A. Conformer 2*

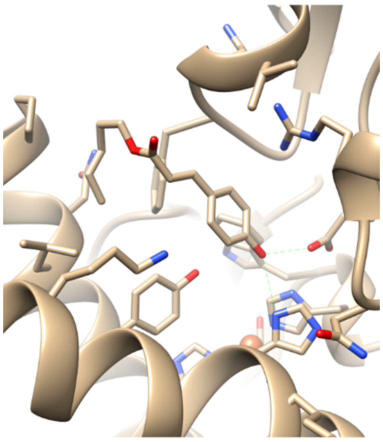

***FS 9** Predicted binding mode of compound **7** to SOD-B.*

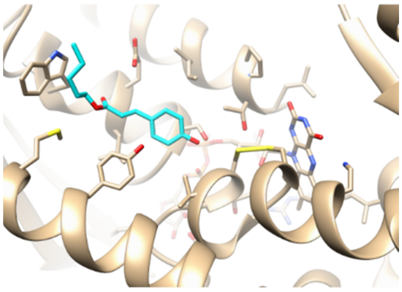

***FS 10** Predicted binding mode of compound **7** to TR. Conformer 1*

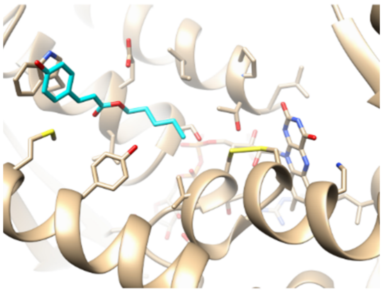

***FS 11** Predicted binding mode of compound **7** to TR. Conformer 2*

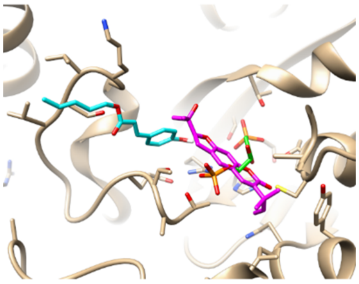

***FS 12** Predicted binding mode of compound **7** to GAPDH (NAD depleted). Conformer 1*

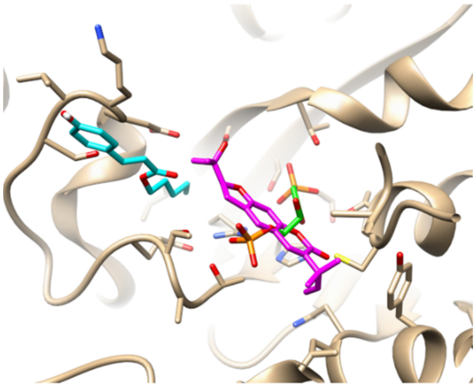

***FS 13** Predicted binding mode of compound **7** to GAPDH (NAD depleted). Conformer 2*

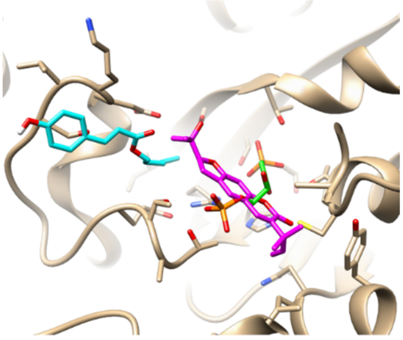

***FS 14** Predicted binding mode of compound **7** to GAPDH (NAD depleted). Conformer 3*

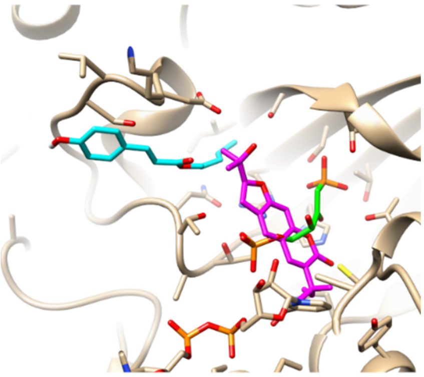

***FS 15** Predicted binding mode of compound **7** to GAPDH with NAD*

References

1. Khatkar, A.; Nanda, A.; Kumar, P.; Narasimhan, B. Synthesis, antimicrobial evaluation and QSAR studies of *p*-coumaric acid derivaties. *Arab J Chem*. **2017**, 10, 3804-3815.
2. Allegretta, G.; Weidel E.; Empting M.; Hartmann R. W. Catechol-based substrates of chalcone synthase as a scaffold for novel inhibitors of PqsD. *Eur J Med Chem*. **2015**, 90, 351–359.
3. Hixson, J. L.; Sleep, N. R.; Capone, D. L.; Elsey, G. M.; Curtin, C. D.; Sefton, M. A.; Taylor, D. K. Hydroxycinnamic Acid Ethyl Esters as Precursors to Ethylphenols in Wine. *J. Agric. Food Chem*. **2012**, 60(9), 2293–2298.
4. Hosseini, R.; Moosavi, F.; Rajaian, H.; Silva, T.; Silva, D. M.; Soares, P.; Saso, L.; Edraki, N.; Miri, R.; Borges, F.; Firuzi, O. Discovery of neurotrophic agents based on hydroxycinnamic acid scaffold. *Chem Biol Drug Des*. **2016**, 88(6), 926–937.
5. Nishimura, K.; Takenaka, Y.; Kishi, M.; Tanahashi, T.; Hiromi Yoshida, H.; Okuda, C.; Mizushin, Y. Synthesis and DNA Polymerase α and β Inhibitory Activity of Alkyl *p*-Coumarates and Related Compounds. *Chem Pharm Bull*. **2009**, 57(5), 476–480.

6. Patel R.B.; Doshi, A. V. Synthesis and study of a new homologous series of Cis Cinnamate Esters of Mesogens: n-Hexyl-p-(p / -n-alkoxy cinnamoyloxy) Cinnamates. *Der Pharma Chemica*. **2011**, 3(1), 338–348.
7. Kim, K. D.; Min, H. S.; Yum, E. K.; Ok, O. S.; Ju, Y. W.; Chang, M. S. Melanogenesis Inhibition by Mono-hydroxycinnamic Ester Derivatives in B16 Melanoma Cells. *Bull Korean Chem Soc*. **2010**, 31(1), 181–184.
